# The effect of calcium hydroxide on the antibiotic component of Odontopaste® and Ledermix® paste

**DOI:** 10.1111/iej.12021

**Published:** 2012-11-27

**Authors:** M Athanassiadis, N Jacobsen, K Nassery, P Parashos

**Affiliations:** 1 Australian Dental ManufacturingBrisbane, Qld, Australia; 2 Private PracticeDulwich, SA, Australia; 3 Melbourne Dental SchoolUniversity of Melbourne, Melbourne, Vic., Australia

**Keywords:** calcium hydroxide, clindamycin hydrochloride, demeclocycline calcium, demeclocycline hydrochloride, Ledermix® paste, Odontopaste®

## Abstract

**Aim:**

To investigate the chemical interaction of calcium hydroxide with the antibiotics demeclocycline calcium in Ledermix® Paste and clindamycin hydrochloride in Odontopaste®.

**Methodology:**

Validated methods were developed to analyse the interaction of calcium hydroxide in two forms, Pulpdent® and calcium hydroxide powder, with the two antibiotics. High-performance liquid chromatography (HPLC) was used to analyse the mixed samples of the pastes and calcium hydroxide. The concentration of demeclocycline calcium over 0-, 1-, 18-, 24-, 72-h and 7-day time-points was determined. The concentration of clindamycin hydrochloride over 1-, 6-, 24-, 72-h and 7-day time-points was determined. All tests with HPLC involved testing of the standard in duplicate alongside the samples. Linearity, precision and specificity of the testing procedures and apparatus were validated. Descriptive statistics are provided.

**Results:**

The antibiotics in both Odontopaste® and Ledermix® Paste were affected by the addition of calcium hydroxide. When mixed with calcium hydroxide powder, Odontopaste® had a 2% loss of clindamycin hydrochloride over 7 days, but when mixed with Pulpdent®, there was a 36% loss over 7 days. Ledermix® Paste showed an 80% loss of demeclocycline calcium over 7 days when mixed with calcium hydroxide powder and a 19% loss when mixed with Pulpdent® over the 7-day period.

**Conclusion:**

The addition of calcium hydroxide to Odontopaste® or Ledermix® Paste results in reductions of the respective antibiotic over a 7-day time period.

## Introduction

Apical periodontitis will develop if there are sufficient numbers of microorganisms present within the root canal system (Kakehashi *et al*. 1965, Möller *et al*. 1981). The use of an intracanal medicament is considered to be essential to maximize the overall success rate of root canal treatment, and it is apparent that calcium hydroxide is currently the most effective medicament for reducing the presence of intracanal bacteria (Byström *et al*. 1985, Stuart *et al*. 1991). This is due to its high pH in an aqueous solution creating an unfavourable environment for the survival and growth of microorganisms within the root canal system (Heithersay 1975).

Ledermix® Paste (Haupt Pharma GmbH, Wolfratshausen, Germany) is a commercially available intracanal medicament that has both an antibiotic component (demeclocycline calcium, which is a tetracycline derivative) and a steroid component (triamcinolone acetonide) in its standard formulation. The presence of the steroid component assists in endodontic treatment by decreasing post-operative pain (Ehrmann *et al*. 2003) and exerting an anticlastic effect on the hosts' cells to prevent inflammatory root resorption (Pierce & Lindskog 1987, Pierce *et al*. 1988, Chen *et al*. 2008). The antimicrobial effect of the demeclocycline calcium has been shown to be inferior to that of calcium hydroxide (Plutzer 2009).

The use of a 50 : 50 mixture of Ledermix® Paste and calcium hydroxide paste has been advocated to obtain the benefits of both an effective antimicrobial agent and an anti-inflammatory agent (Abbott *et al*. 1989a, Taylor *et al*. 1989). However, recent research has shown that in this ratio, the amount of triamcinolone acetonide available is far less than was previously demonstrated, and it may not be of any therapeutic value (Athanassiadis *et al*. 2011).

Similarly, the amount of available triamcinolone acetonide in Odontopaste® is greatly reduced when it is combined 50 : 50 with calcium hydroxide (Athanassiadis *et al*. 2011). The antimicrobial efficacy of demeclocycline calcium and clindamycin hydrochloride within the root canal system is similar (Mohammadi & Abbott 2009a).

All antibiotics have a preferred pH range in which stability is optimal (Florence & Attwood 2011). The use of calcium hydroxide with Odontopaste® and Ledermix® Paste increases the pH of both pastes. This may result in a suboptimal pH range in terms of stability of the antibiotic component.

To date, there are no published data investigating the effects of calcium hydroxide on the antibiotic component of either Ledermix® Paste or Odontopaste® when these products are combined in a 50 : 50 ratio with calcium hydroxide. The aim of this study was to investigate this using validated chemical analysis.

## Materials and methods

All tests were carried out independently by an external laboratory licensed by the Australian Therapeutic Goods Administration (TGA) and with Good Laboratory Practice certification.

### Materials

The materials used included Odontopaste®, Ledermix® Paste, Pulpdent® (Pulpdent Corporation, Watertown, MA, USA) and British Pharmacopoeia (BP)-grade calcium hydroxide powder (Lehmann & Voss & Co, Hamburg, Germany). Prior to use, all batches were tested for compliance with manufacturers' claims. Overage is utilized in Odontopaste®'s manufacture, and therefore, the amount of clindamycin hydrochloride was expected to be greater than the labelled amount to compensate for loss of the active ingredients during manufacturing.

### Method validation

The demeclocycline found in Ledermix® Paste is the calcium salt. Therefore, the method utilized throughout the study allowed for the conversion of the calcium salt to the hydrochloride salt, enabling successful validation. In all instances, the reference to demeclocycline found in Ledermix® Paste is demeclocycline calcium converted to the hydrochloride salt. High-performance liquid chromatography (HPLC) was utilized to analyse the mixed samples of paste with calcium hydroxide.

The validation parameters and the criteria for assessment of the results were chosen to conform to the following protocols:

- The International Conference on Harmonisation of Technical Requirements for Registration of Pharmaceuticals for Human Use (ICH) – Validation of Analytical Procedures: Text and Methodology as adopted by the TGA, Australia (European Medicines Agency 2006).
- Association of Analytical Communities (AOAC)-Single Laboratory Validation Acceptance Criteria, 2009 (AOAC 2009).

These form the basis of the ‘acceptance criteria’ of the chemistry testing undertaken.

The validation parameters within this protocol as per the ICH guidelines are linearity, range, precision, specificity and accuracy. Two HPLC machines were utilized: a Varian Prostar (Agilent Technologies, Santa Clara, CA, USA) and an Agilent 1200 (Agilent Technologies).

Ledermix® Paste was processed using an extraction of 0.1 mol L^−1^ HCl. The extract was then analysed via HPLC utilizing an octyldecylsilyl (C18) column with UV detection at 267 nm. The mobile phase consisted of a mixture of disodium phosphate solution and dimethylformamide (DMF).

Clindamycin hydrochloride was extracted from Odontopaste® using a mobile phase of 45% acetonitrile and 55% of a 0.68% solution of potassium dihydrogen orthophosphate adjusted to pH 7.5 with a 25% w/v solution of potassium hydroxide. The extract was analysed via HPLC utilizing Hypersil BDS-C18 columns with UV detection at 210 nm.

*Linearity* was tested to determine the ability to obtain test results that are directly proportional to the concentration of the analyte in the sample. A stock solution of demeclocycline hydrochloride was prepared using 40.71 mg/100 mL demeclocycline hydrochloride. For clindamycin hydrochloride, a 220 mg/100 mL stock solution was utilized. Dilutions were taken of this stock solution, and these test solutions were then analysed. The results were utilized to prepare a calibration curve. The linear calibration curve was then utilized to estimate the ‘measured concentration’. The coefficient of determination (*R*^2^) was calculated as 0.9998 for the demeclocycline hydrochloride plot and 0.9984 for the clindamycin hydrochloride plot. Both exceeded the acceptance criteria (*R*^2^ ≥ 0.99) as indicated by the TGA, ICH Guidelines and the AOAC. In addition, for each point on the calibration curve, the calibration factor was determined and the relative standard deviation (RSD) was determined. These were analysed and indicated a random pattern with a mean of 0.00. This met the acceptance criteria requirements as indicated by the TGA ICH Guidelines and the AOAC and indicated that no systematic trend was present.

*Systematic precision* was determined by analysing six different vials of an 80 ppm demeclocycline hydrochloride standard and six different vials of 1100 ppm clindamycin hydrochloride. The systematic precision met the required acceptance criteria as per the AOAC of <2% RSD for peak area and <2% RSD for retention times.

*Method precision* was determined by preparing six test solutions utilizing the reagents to extract the clindamycin hydrochloride from the Odontopaste® samples and demeclocycline calcium (converted to demeclocycline hydrochloride) from the Ledermix® Paste samples. The samples were analysed in duplicate through the HPLC. It was found that every sample indicated an approximate 5.6% clindamycin hydrochloride in Odontopaste® and 3.0% concentration of demeclocycline hydrochloride in Ledermix® Paste. The method precision met the required acceptance criteria as per the AOAC guidelines; the standard deviation of 0.43% for clindamycin hydrochloride and 0.69% for demeclocycline hydrochloride were <2%.

*Intermediate precision* was performed in two different ways. The first was by using two different analysts, two different instruments and performing the tests on two different days. The HPLC Varian Prostar was used on 1 day by one operator to analyse six different samples prepared as above. The standard deviation was 0.69% for demeclocycline hydrochloride and 0.55% for clindamycin hydrochloride. Secondly, the HPLC 6 Agilent 1200 was utilized with a different operator, and the same tests performed on a different day. The standard deviation was 0.03% for demeclocycline hydrochloride and 0.21% for clindamycin hydrochloride. Both methods for both antibiotics achieved a standard deviation of <2%, thereby conforming to the required acceptance criteria as stipulated by the AOAC guidelines.

*Specificity* ensures that the analyte alone, free from the effects of the matrix, is being measured. This can be performed by analysing a sample of the placebo where no active substances are present. For Ledermix® Paste, to avoid a possibility of an incorrect placebo being utilized (Athanassiadis *et al*. 2011), a sample of Ledermix® Paste was spiked with additional demeclocycline hydrochloride and a percentage of the sample was utilized to bring the theoretical concentration within range. A recovery of 98.18% was found, which complied with the validation criteria. For Odontopaste®, a placebo was produced and then spiked with clindamycin hydrochloride. A recovery of 98.0% was found, and this complied with the validation criteria stipulated by the AOAC (92–105% for 1% of the analyte, 95–102% for 10% of the analyte).

*Accuracy and spike recovery* were investigated for demeclocycline hydrochloride in Ledermix® Paste using a number of samples spiked with different levels of demeclocycline hydrochloride. Nine individual determinations were performed incorporating three replicate spikes at three different concentration levels of 60, 80 and 100 ppm. Duplicate injections were carried out. The percentage recovery of the spiked samples, that is, the comparison of the calculated amount to the actual amount indicated an average of 100.15% recovery which was within the acceptance criteria for method validation (92–105%) as stipulated by the AOAC guidelines.

For clindamycin hydrochloride in Odontopaste®, five different clindamycin hydrochloride-spiked samples of the placebo paste were utilized. Five different spikes were used on five different samples. The final spiked concentrations tested were 49.6, 98.4, 198.4, 233.7 and 307.1 ppm. The average percentage recovery of the spiked samples was 99.94%, which conformed to the acceptance criteria (92–105%) as stipulated by the AOAC guidelines. The methods between the two pastes were slightly different to accommodate the lack of placebo for Ledermix® Paste.

The method for the determination of demeclocycline calcium from Ledermix® Paste and clindamycin hydrochloride from Odontopaste® was thus validated.

### Testing of starting materials

Odontopaste®, Ledermix® Paste, Pulpdent® and calcium hydroxide were tested by HPLC within 2 weeks prior to commencement of the main testing to confirm manufacturers' specifications. The standard was injected in duplicate alongside the samples, and all results had a difference of <3% between the standards (Table 1).

**Table 1:** Testing of starting materials. All materials complied with manufacturers' specifications

Starting materials	Results
Ledermix® paste	3.0% Demeclocycline hydrochloride
Odontopaste®	5.6% Clindamycin hydrochloride
Pulpdent® paste	40% Calcium hydroxide
Calcium hydroxide powder	98.5% Calcium hydroxide

Odontopaste® and Ledermix® Paste were tested for the initial concentration of clindamycin hydrochloride and demeclocycline hydrochloride, respectively. Pulpdent® was tested for its amount of calcium hydroxide, and the calcium hydroxide powder was tested for purity.

### Testing of combinations

The following products were mixed in a 50 : 50 ratio by weight.

1. Ledermix® Paste with Pulpdent® paste.
2. Ledermix® Paste with calcium hydroxide powder BP.
3. Odontopaste® with Pulpdent® paste.
4. Odontopaste® with calcium hydroxide powder BP.

The ingredients were mixed in a closed container for 3 min, to ensure that a homogenous mix was obtained because large volumes of the pastes were utilized. This did not reflect the mixing times in the clinic typically of <30 s; however, for such a large volume of paste, it was felt a longer mixing time was justified to improve the homogeneity. Following mixing, the samples were then subsampled at various time-points to determine the amount of demeclocycline hydrochloride in Ledermix® Paste and clindamycin hydrochloride in Odontopaste®. The differing time-point testing between the two pastes was as a result of the laboratories' staffing schedules.

For the results, no statistical analysis was carried out because only one time-point trial was undertaken. Even though validated methods were developed, which improve the accuracy of the testing, it remained appropriate that only descriptive analysis be reported.

## Results

The results indicated that the antibiotics in both Odontopaste® (Table 2) and Ledermix® Paste (Table 3) were affected by the addition of calcium hydroxide. Odontopaste® was less affected when mixed with calcium hydroxide powder with only a 2% loss over 7 days; however, when mixed with Pulpdent®, there was a 36% loss over 7 days. Ledermix® Paste was also affected when mixed with calcium hydroxide powder with an 80% loss over 7 days and a 19% loss when mixed with Pulpdent® over the same 7-day period. During analysis, there were time-points where the concentration of the clindamycin and demeclocycline rose slightly. This may be due to the effect on homogeneity from mixing the two components or gaseous loss of components due to interactions with calcium hydroxide.

**Table 2:** The time-point results of Odontopaste® mixed with calcium hydroxide powder and Pulpdent® in a 50 : 50 ratio. The percentage loss of clindamycin hydrochloride was calculated on 50% of the premix percentages

Addition of 50% Calcium hydroxide powder							

Compound	Premixing	0 h	1 h	6 h	24 h	3 days	7 days
Clindamycin%	5.6	2.83	2.85	2.89	2.87	2.82	2.75
% Loss		0	0	0	0	0	2

Addition of 50% Pulpdent® (40% Calcium Hydroxide in an aqueous base)							

Compound	Premixing	0 h	1 h	6 h	24 h	3 days	7 days
Clindamycin%	5.6	2.73	2.42	2.31	2.69	1.95	1.80
% Loss		3	14	18	4	30	36

**Table 3:** The time-point results of Ledermix® Paste mixed with calcium hydroxide powder and Pulpdent®. The percentage loss was calculated on 50% of the premix percentages

Addition of 50% Calcium hydroxide powder							

Compound	Premixing	0 h	1 h	18 h	24 h	3 days	7 days
Demeclocycline%	3.0	1.35	1.53	0.76	0.57	1.27	0.30
% Loss		10	0	49	62	15	80

Addition of 50% Pulpdent® (40% Calcium hydroxide in an aqueous base)							

Compound	Premixing	0 h	1 h	18 h	24 h	3 days	7 days
Demeclocycline%	3.0	1.50	1.35	1.53	1.52	1.67	1.21
% Loss		0	10	0	0	0	19

## Discussion

Both Odontopaste® and Ledermix® Paste contain antibiotics to which *Enterococcus faecalis* exhibits resistance. However, the concentration of the antibiotic within both pastes exceeds the typical minimum inhibitory concentration (MIC) of *E. faecalis* several thousandfold (Rossi-Fedele & Roberts 2007). Therefore, resistance to the antibiotic around and within the paste does not present a contraindication to their use. The main determining factor for the efficacy of the paste against *E. faecalis* is not the resistance to the antibiotic but rather the biofilm form in which the bacteria are present within the root canal (Lima *et al*. 2001, Plutzer 2009). It is this biofilm that reduces the antibacterial properties of the formulation. In addition, the loss of the antibiotic may allow for the direct colonization of the paste by bacteria. This may be an issue as a result of the breakdown of the antibiotic by calcium hydroxide and the loss of alkalinity of the combined paste over time.

It is important to note that the demeclocycline in Ledermix® is the calcium salt. Previous diffusion studies involved the addition of tritium-labelled tetracycline and scintillation chemistry to determine the rate of diffusion of demeclocycline from Ledermix® Paste into dentine (Abbott *et al*. 1989b). However, the salt-type of the tritium-labelled tetracycline is unknown, and there is the possibility that the two forms of tetracycline differ in solubility. This would affect the results of the testing. For tetracycline, calcium salts are less soluble than hydrochloride salts (Sarfaraz 2007). The advantage of calcium salts is an increase in the shelf life of the product as a result of low solubility (Gould 1986).

The pKa properties of the antibiotic salts are also important. When the pKa of a salt is two units lower than the pH of the environment, complete dissociation of the compound occurs. In this case, the antibiotic will be present. The hydrochloride salts have low pKa and hence tend to be fully dissociated at neutral pH allowing for better availability of the antibiotic. A calcium salt of a tetracycline is more complex as tetracyclines are considered amphoteric drugs displaying both basic and acidic characteristics. In addition, tetracyclines behave as chelating agents binding strongly to calcium (Florence & Attwood 2011). Therefore, the addition of calcium hydroxide raises the pH, and, depending on the pKa of the antibiotic salt, this will in turn have an effect on the availability of the antibiotic.

The addition of calcium hydroxide to the pastes also changes the equilibrium of the ionization of the salt. The process is referred to as the common ion effect (Silberberg 1996). The ionization equilibrium is shifted towards the unionized salt due to the excess calcium ions. This may reduce the availability of the antibiotic. Because clindamycin hydrochloride is not present as a calcium salt, its dissociation equilibrium is not affected (Gould 1986). Further research is required to determine the effect of calcium hydroxide on demeclocycline calcium.

The steroid component of Odontopaste® and Ledermix® Paste, triamcinolone acetonide, is broken down rapidly by calcium hydroxide (Athanassiadis *et al*. 2011). The breakdown products are unknown. The present study indicates that each antibiotic in each paste also breaks down, albeit at a lower rate. The breakdown products are also unknown. Degraded tetracycline, from expired medication, is a known cause of Fanconi's Syndrome – a disease of the proximal renal tubules (Frimpter *et al*. 1963). It is unknown whether demeclocycline behaves similarly although renal impairment is a known side effect of larger oral doses of demeclocycline (Zietse *et al*. 2009). There is no reported toxicity due to expired clindamycin hydrochloride medication. A major breakdown product of clindamycin hydrochloride is lincomycin hydrochloride, another antibiotic (Oesterling 1970).

The preferred timing of action of the steroid, and where it produces its most clinically significant effect of reducing pain and inflammation, is within the first 3 days following the application of the paste (Mohammadi & Abbott 2009b). The antibiotic, however, is expected to perform over a longer period making the requirement for stability of the antibiotic over a longer time-frame more important.

Although this study reports the results of only one test, from which it would be difficult to draw definitive outcomes, the data indicate that a substantial reduction in the antibiotic concentration in both Ledermix® Paste and Odontopaste® occurs when mixed with calcium hydroxide. Both pastes suffered breakdown of their antibiotics within 7 days when mixed with Pulpdent®. The clindamycin in Odontopaste® was more stable than the demeclocycline in Ledermix® when mixed with calcium hydroxide powder over the same period. The rate of loss of antibiotic over time was not consistent and indicates the importance of the role of mixing. The loss is difficult to predict in a quantified manner. Furthermore, whilst the clinical impact of the reduction in the effect of the antibiotics could be correlated to a second method such as agar diffusion tests on specific microorganisms, the focus of this work has been to alert clinicians of the potential problems associated with the mixing of intracanal medicaments without consideration of the chemistry of such mixtures.

In addition, the effect of the additional calcium hydroxide on the homogeneity of the pastes is unknown and may explain some of the variations in the results. There was no need to progress the testing more than the 7-day period as the level of destruction of the antibiotic indicated that long-term stability of the mixed product was unfavourable.

This study did not attempt to validate the mixing. Although this represents a shortcoming in the method, it does represent a true reflection of the clinical application of the 50 : 50 regimen.

The mixing of additional calcium hydroxide with Odontopaste® or Ledermix® Paste may be of benefit in terms of antibacterial properties, if there is a proven synergistic relationship of the antibiotic and calcium hydroxide. Studies to determine this have been equivocal. For *Lactobacillus casei* and *Streptococcus mutans,* the 50 : 50 mixture with Ledermix® Paste has been shown to be marginally more effective than each individual paste (Taylor *et al*. 1989). Another study indicated that the addition of 25% Calxyl to Ledermix® Paste reduced the antimicrobial properties against *Streptococcus sanguis* and *Staphylococcus aureus* (Seow 1990). Biofilm forms of *E. faecalis* have been found to be resistant to the antibiotics in Odontopaste® and Ledermix® (Plutzer 2009). When calcium hydroxide in the form of Pulpdent® was mixed 50 : 50 with the paste, it was found that the effects were comparable to calcium hydroxide alone in that the *E. faecalis* biofilm was destroyed (Plutzer 2009). Therefore, either the antibiotic and calcium hydroxide mixed 50 : 50 does have an effect, or a lower percentage of calcium hydroxide in an aqueous/polyethylene glycol liquid base is effective against *E. faecalis* in biofilms. Importantly, no studies have shown the MIC of calcium hydroxide in biofilms within a root canal.

In terms of chemistry, the solubility product (*K*_sp_) of calcium hydroxide is 6.5 × 10^−6^ at 25 °C resulting in 1.48 g L^−1^ at 25 °C (including the common ion effect), decreasing as the temperature increases (Silberberg 1996). A paste with 40% calcium hydroxide in an aqueous base equals 400 g of calcium hydroxide in 1 kg of paste (w/w). Assuming minimal gelling agent is added, there would be approximately 600 g of water to 400 g of calcium hydroxide. The solubility of calcium hydroxide in water is 1.48 g L^−1^ or 0.888 g in 600 mL of water (0.148%), assuming specific gravity of water is one. Anything above 0.148% of calcium hydroxide will result in the maximum molar concentration in solution (assuming total dissociation). This puts into perspective the importance of calcium hydroxide in the paste. A 20% calcium hydroxide paste will provide the same results in terms of chemistry as a 40% calcium hydroxide paste with the advantage of the 40% paste being that the ability to replace depleted calcium hydroxide in solution is greater due to the greater reservoir. Whether or not this is clinically significant in a paste whose use is commonly confined to several weeks needs to be determined.

This may, however, explain why a 50 : 50 ratio of calcium hydroxide in Odontopaste® or Ledermix® Paste manages to provide similar levels of effectiveness against *E. faecalis* in biofilm form. The effect of polyethylene glycol as a wetting agent or surfactant has also not been investigated. This too may enhance the ability of calcium hydroxide to exert an effect on biofilms by making them more prone to reacting with hydroxyl ions. There are several proprietary calcium hydroxide formulations based on polyethylene glycol (Leonardo *et al*. 1993).

The relevance of the pH of pastes is also complex and often misunderstood. The fact that both Odontopaste® and Ledermix® Pastes consist of differing mixed-solvents of water and polyethylene glycol makes pH comparisons, in a strict sense, difficult. In addition, the pH of calcium hydroxide in an aqueous base cannot be compared with the pH of calcium hydroxide in a polyethylene glycol, or mixed-solvent base, as the pH scales are inherently different, the autoprotolysis constant is different and the junction potential of the glass electrodes in the pH meters typically used to make the measurement, changes (Tindall 2002). This is an acknowledged issue with pH measures between substances in different solvents (Rondinini 2002, Himmel *et al*. 2010). Each unique solvent has its own pH range, and the measures can only used for comparison within the same solvent (Tindall 2002). As a result, manufacturers of polyethylene glycol note the pH as a measure of an aqueous dilution of polyethylene glycol, typically 5–10%. Polyethylene glycol, measured in this manner, is considered mildly acidic (Dow Chemical Company 2011).

The effect of calcium hydroxide in a 50 : 50 ratio with Odontopaste® and Ledermix® goes beyond the effect on the antibiotic. It asks many questions regarding the understanding of the chemistry of the hybrid paste. To dissect the drug interactions in more depth to identify the breakdown products involves complex chemistry and represents data that even manufacturers do not possess. Further research in these areas is required.

## Conclusions

The mixing of calcium hydroxide in the form of raw powder, or as Pulpdent®, results in the breakdown of the antibiotics in Odontopaste® and Ledermix® Paste. In applications >7 days, there would be concern regarding the levels of antibiotic present and its therapeutic benefit. The continued use of the 50 : 50 protocol of mixing a form of calcium hydroxide with Odontopaste® or Ledermix® Paste should be reconsidered with further research to gain a better understanding of the chemistry involved with the 50 : 50 protocol.
